# Thickness of the particle-free layer near charged interfaces in suspensions of like-charged nanoparticles

**DOI:** 10.1039/d1sm00584g

**Published:** 2021-05-27

**Authors:** Dominik Kosior, Manuchar Gvaramia, Liam R. J. Scarratt, Plinio Maroni, Gregor Trefalt, Michal Borkovec

**Affiliations:** Department of Inorganic and Analytical Chemistry, University of Geneva, Sciences II 30 Quai Ernest-Ansermet 1205 Geneva Switzerland michal.borkovec@unige.ch

## Abstract

When a suspension of charged nanoparticles is in contact with a like-charged water–solid interface, next to this interface a particle-free layer is formed. The present study provides reliable measurements of the thickness of this particle-free layer with three different techniques, namely optical reflectivity, quartz crystal microbalance (QCM), and direct force measurements with atomic force microscopy (AFM). Suspensions of negatively charged nanoparticles of different size and type are investigated. When the measured layer thickness is normalized to the particle size, one finds that this normalized thickness shows universal inverse square root dependence on the particle volume fraction. This universal dependence can be also derived from Poisson–Boltzmann theory for highly asymmetric electrolytes, whereby one has to assume that the nanoparticles represent the multivalent coions.

## Abstract 2

When a suspension of charged nanoparticles is in contact with a like-charged water–solid interface, next to this interface a particle-free layer is formed.

## Graphical abstract

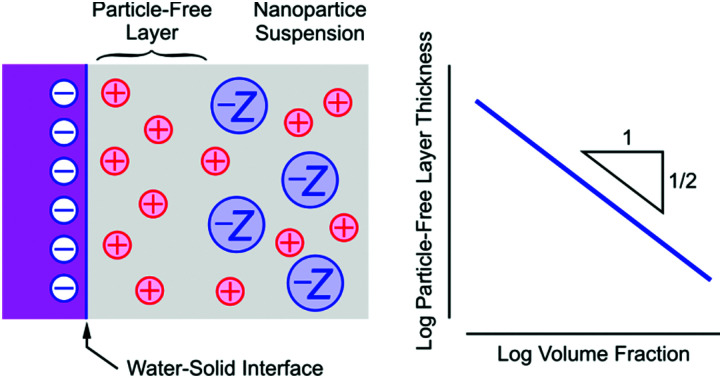

## Introduction

Interactions involving concentrated suspensions of charged colloidal nanoparticles and interfaces were of substantial interest recently.^1–4^ Particular attention was devoted to systems where the interfaces and the nanoparticles were like-charged, whereby avoiding deposition of the particles to the substrate. The interactions between interfaces were mostly probed with various techniques in the slit geometry, including interferometry,^5,6^ optical tweezers,^1^ or the colloidal probe technique based on atomic force microscopy (AFM).^2,4,7–10^ The interesting observation made in these studies was that the typical force profiles were oscillatory. The corresponding wavelength was found to decrease with increasing particle concentration, thereby reflecting a liquid-like structure of the nanoparticle suspension. The existence of such oscillatory profiles was also confirmed in nanoparticle suspensions near isolated like-charged interfaces with X-ray^11^ and neutron reflectivity.^12^ Similar oscillatory profiles were also observed in concentrated polyelectrolyte^13–16^ and micellar solutions.^17,18^

Another important aspect concerning interactions in suspensions of charged nanoparticles and like-charged interfaces is the presence of a particle-free layer next to the interface. The existence of such a layer appears to be rather obvious due to the electrostatic repulsion between the like-charged interface and the nanoparticles. However, experimental evidence concerning this layer is relatively scarce. Its existence has been confirmed in the slit geometry by direct force measurements in nanoparticle suspensions^9,10^ and polyelectrolyte solutions.^19,20^ The presence of a particle-free layer was also suggested in nanoparticle suspensions in contact with an isolated interface based on X-ray^11^ and neutron reflectivity.^12^ Finally, a recent quartz crystal microbalance (QCM) study of charged latex particle suspensions near a like-charged interface advocated the presence of a similar particle-free layer too.^21^ Some of these studied reported that the thickness of this particle-free layer decreases with increasing particle concentration.

Several theoretical studies equally suggested the presence of this particle-free layer. The most comprehensive investigations were presented by Gonzalez-Mozuelos *et al.*^22,23^ The authors use an integral equation theory combined with the hypernetted chain closure to calculate concentration profiles in charged nanoparticle suspensions next to charged interfaces. These calculations predict such particle-free layers with a thickness that substantially exceeds the particle diameter. More recently, the existence of such particle-free layers was also shown with density functional theory.^24^ These theories also suggest an oscillatory concentration profile near the like-charged interface whereby the first peak is the most pronounced one. One should note that this peak is located at somewhat larger distances than the thickness of the particle-free layer.

These experimental and theoretical studies thus strongly suggest the existence of such particle-free layers, but they do not provide additional understanding of its generic properties. In particular, the dependence of the thickness of the particle-free layer on the particle concentration has been hardly addressed.

The goal of this article is to advance our understanding of this particle-free layer, and put forward its generic features. In particular, we show that the extension of the classical Poisson–Boltzmann (PB) theory to highly asymmetric electrolytes predicts the existence of such a layer, and also explains why this layer features a well-defined thickness. Subsequently, we report on measurements of the thickness of this particle-free layer in different nanoparticle suspensions with three different experimental techniques. Besides the established direct force measurements^4,9,25^ and QCM technique,^21,26,27^ we demonstrate that the thickness of this layer can be also measured with optical reflectivity.^28,29^ We further argue that the characteristic separation between two interfaces, as observed in direct force measurements, corresponds to twice the layer thickness for an isolated interface.

### Poisson–Boltzmann model

To estimate the layer thickness theoretically, we use one-dimensional Poisson–Boltzmann (PB) model for a 1 : *Z* electrolyte. We consider a negatively charged interface, thus like-charged to the multivalent anions, and oppositely charged to the monovalent cations. Here we are interested in highly asymmetric electrolytes, whereby the multivalent anions represent the nanoparticles. In this situation, the multivalent co-ions are strongly repelled from the interface, thereby creating a layer that contains monovalent couterions only. As we shall see, this layer corresponds precisely to the particle-free layer discussed in the introduction.

The PB model is normally formulated in terms of a differential equation for the total electric potential *ψ*(*x*), where *x* is the distance normal to the interface, and reads1
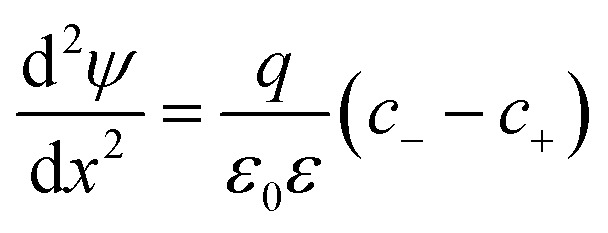
where *q* is the elementary charge, *ε*_0_ the permittivity of vacuum, *ε* the dielectric constant of water. Note that the electric potential originates from all ion species and the charged wall(s). The number concentration profiles are given by the following expressions, namely for the multivalent anions

2 *c*_−_ = *ce*^*βqZψ*^

and for the monovalent cations

3 *c*_+_ = *Zce*^−*βqψ*^

where *Z* is the valence of the multivalent anion (*Z* > 0) and *β* = 1/(*kT*) whereby *T* is the absolute temperature and *k* the Boltzmann constant. We suppose a temperature of 25 °C and use *ε* = 80 as appropriate for water throughout. The electric potential is assumed to vanish in the bulk, and therefore the concentration *c* of the multivalent anions is equal to the one of the 1 : *Z* salt, while of the one for monovalent cations is given by *Zc* by virtue of electroneutrality. Eqn (1) is then solved in two different geometries numerically.

#### Isolated interface

In this situation, the interface is positioned at *x* = 0 and the electrolyte solution in the half-space *x* > 0. In this situation, the boundary conditions can be specified as follows. We have for *x* → ∞ d*ψ*/d*x* → 0 due to electroneutrality, and we assume that *ψ*(*x*) → 0. The diffuse layer potential has to be specified at the interface, thus *ψ*(0) = *ψ*_dl_. One may also specify the surface charge density, but this parameter is related to the diffuse layer potential by the charge–potential relationship^30–32^

4 *σ* = sgn(*ψ*_dl_)[2*kTε*_0_*εc*(*Ze*^−*βqψ*_dl_^ + *e*^*βqZψ*_dl_^ − 1 − *Z*)]^1/2^

**Fig. 1:**
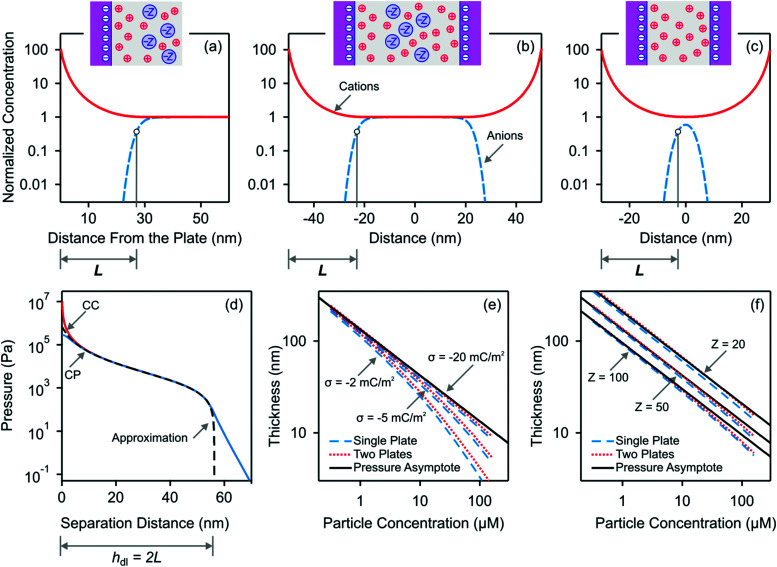
PB calculations of the co-ion-free layer for the 1 : *Z* electrolyte next to negatively charged interfaces. Top rows shows the bulk normalized concentration profiles for (a) a single plate, (b) a wide slit and (c) a narrow slit. The schemes indicate the ionic distributions pictorially. The onset of the co-ion-free layer is defined where the electrical energy of the co-ions attains the value of *kT*. (d) Pressure profile for constant charge (CC) and constant potential (CP) boundary conditions together with the analytical approximation given in eqn (9). Concentration dependence of the thickness *L* of the co-ion-free layer for a single plate and two plates for (e) different surface charge densities and (f) different co-ion valence. Unless otherwise indicated we use *Z* = 50, *c* = 20 μM, and *σ* = −20 mC m^−2^.

Representative concentration profiles normalized to the bulk concentrations are shown in Fig. 1a. For these calculations, we have used the surface charge density *σ* = −20 mC m^−2^, a salt concentration *c* = 20 μM, and co-ion valence *Z* = 50. One observes the formation of a co-ion free layer with thickness of about 27 nm. As one approaches the interface, the concentration of the co-ions decreases extremely rapidly, and this layer is virtually free of the co-ions.

#### Two interacting interfaces

The symmetric slit-geometry involving two identical interfaces is relevant for direct force measurements. We assume that the interfaces are situated at *x* = ±*h*/2, where *h* is the distance between the interfaces, together with the boundary conditions5
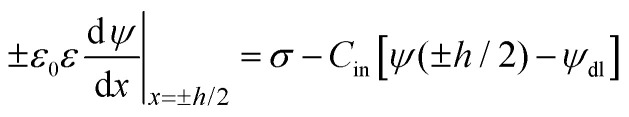
where *C*_in_ is the inner capacitance. The inner capacitance is normally expressed in terms of the regulation parameter defined as6
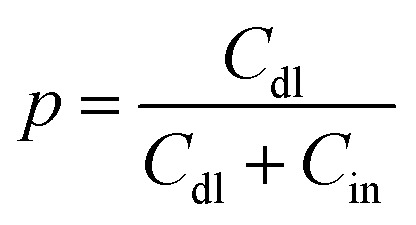
where *C*_dl_ is the diffuse layer capacitance defined as *C*_dl_ = ∂*σ*/∂*ψ*_dl_. The advantage of the regulation parameter is that it assumes simple values for the classical boundary conditions of constant potential (CP, *p* = 0) for constant charge (CC, *p* = 1).

The corresponding concentration profiles are shown in Fig. 1b and c. When the interfaces are sufficiently separated, the concentration profiles near each interface are basically identical to the ones of the isolated interface. This fact is not surprising since the case of the isolated interface is recovered when the separation distance is sufficiently large. When the separation decreases, a co-ion free gap forms at a separation distance that is twice as large as the thickness of the particle-free layer for the isolated interface.

When electric potential profile for a given separation *h* is known, the pressure *Π* between the plates, which is the force per unit area, can be obtained as^30–32^

7 *Π* = *kTc*(*Ze*^−*βqψ*_mp_^ + *e*^*βqZψ*_mp_^ − 1 − *Z*)

where *ψ*_mp_ = *ψ*(0) is the midplane potential. A typical pressure profile is shown in Fig. 1d. At small separations the pressure decreases gradually, while at larger distances more rapidly. The gradual decay occurs as long as the gap is free of co-ions. The rapid decay sets in as soon as the co-ions enter the slit. One may note that the boundary conditions are important at small separations only.

Direct force measurements are typically carried out with larger microparticles, and therefore the measured force can be obtained by means of the Derjaguin approximation^30,32^8
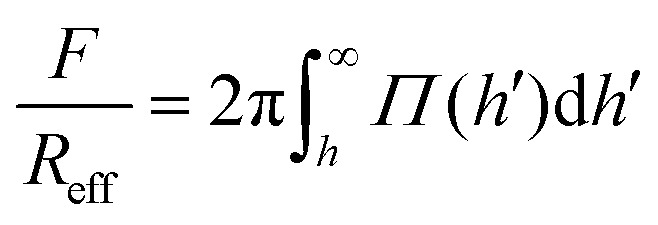
where *R*_eff_ is the effective radius. In the symmetric sphere-sphere geometry, which will be used here, the effective radius is given by *R*_eff_ = *R*/2 where *R* is the radius of the microparticles.

#### Analytical estimates

The pressure profile is known analytically for the salt-free conditions.^33^ For highly asymmetric electrolytes (*Z* ≫ 1), however, this expression must be corrected by the osmotic pressure of the electrolyte and one finds^19^9
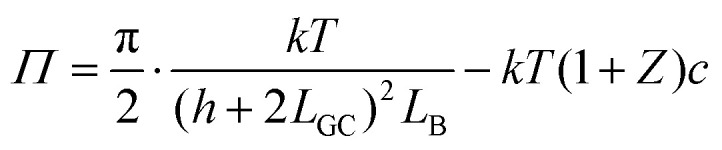
where *L*_GC_ is the Gouy–Chapman length10
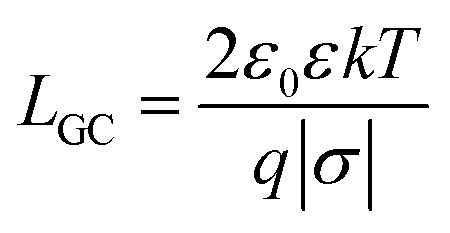
and *L*_B_ is the Bjerrum length given by11
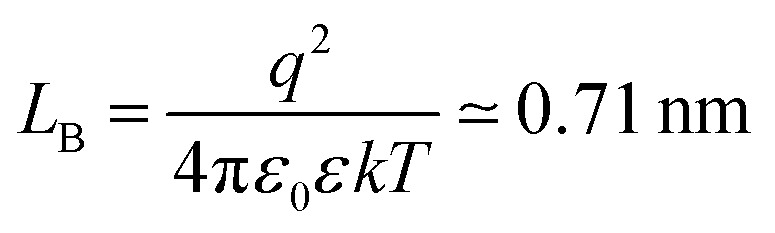
Note that eqn (9) applies only at sufficiently large separation distances, and for this reason this expression is independent of the boundary conditions. The validity of this approximation is illustrated in Fig. 1d. This relation describes the gradual decay at small distances, which occurs within the co-ion free gap. At larger distances, eqn (9) predicts a decay that is steeper than the prediction of the PB theory. The validity of the PB description for interaction forces was demonstrated for multivalent co-ions some time ago,^34^ and more recently for polyelectrolytes and nanoparticles.^9,19^

Eqn (9) is useful to obtain an analytical estimate of the thickness of the salt-free gap through the condition

12 *Π*(*h*_dl_) = 0

One should note that the corresponding gap thickness *h*_dl_ represents twice the thickness of the salt-free layer *L* next to an isolated interface, namely

13 *L* = *h*_dl_/2

To avoid confusion, we will use only the thickness *L* next to the isolated interface in the following, and divide the gap thickness from in the slit geometry by a factor of two. The diffuse layer thickness can be further simplified by assuming that the substrate is highly charged, whereby the contribution from the Gouy–Chapman length in eqn (9) becomes negligible. For *Z* ≫ 1 one finds from eqn (12) the simple expression14
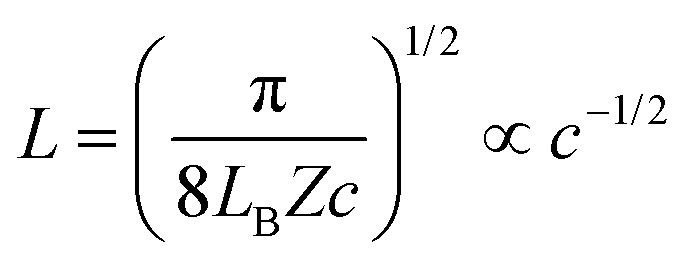
This relation will be referred to as the pressure asymptote. An analogous pressure asymptote was proposed earlier to describe the thickness of charged anionic bilayers in the presence of multivalent coions.^35^ This relation predicts that the layer thickness decreases with an inverse square root of the salt concentration.

The pressure asymptote has been compared with various layer thickness calculations based on the full PB equation for the isolated interface and the slit. Thereby, the layer was defined such that the electrostatic energy of the co-ions is given by one *kT*. The results for the slit geometry were divided by a factor of two as indicated in eqn (13). The comparison of these results is shown in Fig. 1e and f. One observes that the numerical calculations follow the pressure asymptote for highly charged interfaces very well. For weakly charged surfaces, the decrease becomes more substantial. The pressure asymptote depends on the valence of the co-ions and this dependence also agrees with the numerical calculations very well.

One should realize that the PB model relies on a mean-field approximation but is expected to be a reasonable approximation in the region close to the interface, which is dominated by the monovalent counterions only. However, this approximation will fail at larger distances, where the concentration profiles are expected to be oscillatory.

## Experimental

### Nanoparticles

Silica (HS30) and sulfate latex nanoparticle suspensions were purchased from Sigma-Aldrich and Invitrogen, respectively. The used batch of silica particles is similar to the one used in our previous studies,^9,12^ but is not identical to the latter. Latex A is the same as the one used in a previous study by Scarratt *et al.*^9^

Prior to experiments, impurities were removed from the suspensions by ultrafiltration or dialysis. The silica suspension was purified in a stirred cell (Amicon, Millipore) against ultrapure water with regenerated cellulose ultrafiltration discs with a molecular mass cut-off of 5 kg mol^−1^ (Amicon, Millipore). The process was terminated when the conductivity of the filtrate dropped below 40 μS cm^−1^. The latex particles were dialyzed against pure water with a cellulose ester membrane (Repligen) until the conductivity of water dropped below 10 μS cm^−1^. Membranes with a mass cut-off of 0.5 kg mol^−1^ were used for latex A, and for the other latex particles a cut-off of 300 kg mol^−1^ was used. The pH of the purified silica and latex suspensions were 8.3 ± 0.3 and 4.0 ± 0.5, respectively. Based on these observations, we suspect that the latex suspensions are basically salt-free. On the other hand, given the high pH and higher conductivities of the silica suspensions, traces of NaOH originating from the synthesis process might be present.

The particles were characterized with atomic force microscopy (AFM, Cypher, Oxford Instruments) by imaging in amplitude modulation mode with a scan rate of 1 Hz. Cantilevers with tetrahedral silicon tips (AC160TS-R3, Olympus, Japan) were used to record the topographic images in air. Their resonance frequencies were around 300 kHz and their spring constants around 30 N m^−1^. The excitation of the cantilevers was done at their resonance frequencies. Free oscillation amplitudes (FOA) of around 12 nm were used with set points around 75% of the FOA. Particle size distributions were obtained with an image analysis program. The number averaged particle radii and the polydispersity, as expressed in terms of the coefficient of variation (CV), are summarized in Table 1. The values for the silica particles are similar to the ones used by some of us previously,^9,12^ and very close to the ones for a presumably similar HS30 sample.^36^ The measured values for the latex particles agree well with the ones reported by the manufacturer, with the exception of the polydispersities, which we found somewhat larger, especially for the smallest particles. A similar discrepancy is also present for latex A, which was already used in a previous study by Scarratt *et al.*^9^ We suspect that this difference is due to poor contrast in transmission electron microscopy of the very small latex particles that are present in the sample. This technique was used for the particle sizing by the manufacturer and by Scarratt *et al.*^9^

**Table 1:** Sizing and optical properties of the nanoparticles used

	Radius (nm)		CV^b^	RII^c^ (mL g^−1^)
	DLS^a^	AFM^b^		
Silica	8.0	7.8	0.18	0.0674
Latex A	13	9.1	0.33	0.232
Latex B	23	21	0.30	0.235
Latex C	32	28	0.12	0.237
Latex D	54	50	0.10	0.288

a Hydrodynamic radius measured by dynamic light scattering (DLS) in dilute suspension.

b Obtained by image analysis of dried samples with the atomic force microscope (AFM).

c Refractive index increment (RII) measured with an optical refractometer.

The mass concentration of the purified suspensions was determined by drying overnight at 110 °C to constant weight. The densities of the silica and latex nanoparticles were 2.29 g cm^−3^ and 1.06 g cm^−3^, respectively. The former value was as measured by weighing within the suspension (Easy Dyne K20, Krüss) and the latter was reported by the manufacturer. The volume fraction *ϕ* was determined from the mass concentration by assuming ideal mixing. The number concentration *c* can be obtained from the relation15
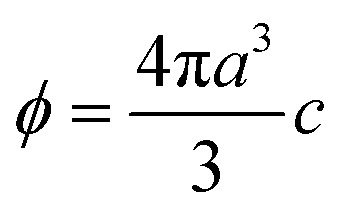
where *a* is the particle radius. The refractive index increment (RII) of the particle suspensions was measured with an automatic refractometer (Abbemat WR/MW, Anton Paar GmbH) and the resulting values are summarized in Table 1.

The nanoparticles were further analyzed with dynamic light scattering (DLS) and electrophoresis in 1.0 mM NaCl solutions. The DLS measurements were performed on a compact goniometer (CGS-3, ALV, Germany) at a scattering angle of 90°, while the electrophoretic mobility was measured with Zetasizer ZS (Malvern). For the silica particles, the measurements were carried out at pH 8.5 at a particle concentration of 1.0 g L^−1^. For the latex particles, pH 4.0 was used. For latex A the particle concentration was 1.0 g L^−1^ and for the other latex samples a concentration range of 0.05–0.20 g L^−1^ was used. The hydrodynamic radius was extracted from the DLS measurements by means of second cumulant analysis and the Stokes–Einstein relation.^30^ The measured radii obtained by DLS are summarized in Table 1 and they are systematically larger than the values obtained by AFM. This discrepancy is expected since the DLS weighs the larger particles more strongly. These results can be further influenced by hydration effects and minor deviations from spherical shape. The electrophoretic mobility was converted to the electrokinetic potential with the theory of O’Brien and White^37^ and to surface charge density with the extension of the Grahame equation to the spherical geometry, which is applicable down to intermediate salt levels.^30^ The respective results are summarized in Table 2. Ultrapure Milli-Q water (Millipore) was used throughout. Unless noted otherwise, the experiments were carried out at room temperature of 21 ± 2 °C.

**Table 2:** Charging characteristics of the nanoparticles used

	Mobility^a^ (×10^−8^ m^2^ V s^−1^)	*ζ*-Potential^a^ (mV)	Charge density^a^ (mC m^−2^)	Charge density^b^ (mC m^−2^)
Silica	−3.3	−70	−12	−13
Latex A	−4.7	−150	−41	−36
Latex B	−3.7	−84	−11	−10
Latex C	−3.8	−96	−14	—
Latex D	−3.8	−86	−11	—

a Electrophoretic mobility together with corresponding electrokinetic *ζ*-potentials and charge densities of the nanoparticles.

b Charge densities of the nanoparticles obtained from force measurements wherever possible.

### Optical reflectivity

These measurements were carried out with silicon wafers (p-type, Silchem) as substrates. The wafers were cut to an approximate squares of 1 cm and treated at 1000 °C for 10–20 minutes to obtain thermally grown silica layer on its surface. The wafer was then cleaned by sonication in ethanol (99.8%, Fluka) for 20 min, dried in a flow of nitrogen, and finally treated in air plasma for 30 min (PDC-32G, Harrick).

The reflectivity signal was measured in a home-built fixed-angle reflectometer. One arm of the reflectometer carries a polarized green diode laser with a wavelength of 532 nm. The wafer is mounted in a stagnation-point flow cell covered with a capped prism, which is separated from the crystal by a spacer. A peristaltic pump is used to pump the suspensions through a vertical bore hole in the prism at a flow rate of 0.5 mL min^−1^. The light beam reflected from the wafer is separated into its perpendicular and normal components by a polarizing beam splitter, and the respective intensities are measured with photodiodes with a lock-in detection scheme. The ratio of these intensities *R* is recorded *versus* the experimental time *t*. Finally, one calculates the reflectometry signal16
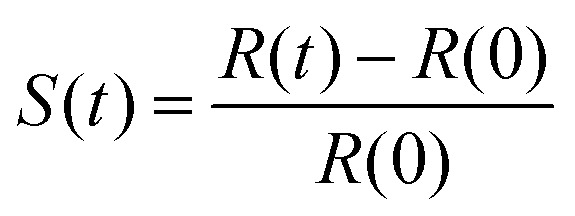
whereby the unknown instrumental constant is eliminated. Further details on this setup are given elsewhere.^28,38^

The precise thickness of the silica layer was determined for each wafer by null ellipsometry (Multiskop, Optrel) in air at a fixed angle of 69° with a laser light source of a wavelength of 633 nm. The ellipsometry data were analyzed with a slab model whereby the respective refractive indices silicon and silica were fixed at 3.85 + 0.02*i* and 1.457, respectively.^39,40^ The resulting thickness of the silica layer was typically in the range of 10–30 nm.

### Quartz crystal microbalance

Quartz crystal sensors were used as substrates (QSX303, Q-Sense). These sensors consist of a quartz oscillator, which is coated with a gold layer of about 100 nm thickness, which is coated on its top with a silica layer of about 300 nm by means of physical vapor deposition. Prior to use, the sensor was cleaned by immersion into solution of sodium dodecyl sulfate (Sigma Aldrich) with a concentration of 20 g L^−1^ for 30 minutes, then by rinsing with pure water, and subsequently dried in a flow of nitrogen. The substrate was then treated with a UV-ozone cleaner (PSD-Pro Series, Novascan) for 20 min, then a drop of a 1 : 1 mixture of H_2_SO_4_ (96%, Carlo Erba Reagents) and H_2_O_2_ (30%, Reactolab SA) was placed on the substrate for 5 min, and finally the sensor was rinsed with pure water and dried in a flow of nitrogen.

Quartz crystal microbalance (QCM, Q-sense E4, Gothenburg, Sweden) with a flow-through cell was used to monitor the frequency shifts Δ*f* and the changes in the dissipation signal Δ*D* for different overtone numbers *n* = 3, 5, 7, 9, 11. The frequency *f* reflects the resonance frequency of the crystal, while the dissipation is defined as *D* = *E*′/(2π*E*) where *E*′ is the energy loss during an oscillation, and *E* is the energy stored in the quartz oscillator. The measured frequency shifts were converted to the normalized frequency shifts Δ*f*/*n*. The crystal is mounted in a thin-layer cell with two eccentric holes that serve as the inlet and outlet. The suspensions are pumped through the cell with a peristaltic pump at a flow rate of 0.5 mL min^−1^. The measurement cell is kept at a constant temperature of 25.0 ± 0.2 °C. More details on similar measurements can be found elsewhere.^21,41^ Further information on the QCM technique is given in appropriate reviews.^42,43^

### Direct force measurements

Silica microparticles (Bangs Laboratories Inc.) with a radius of about 2.5 μm were used as colloidal probes. The microparticles were glued to tipless cantilevers (HQ CSC37, MikroMasch, Tallin, Estonia) by means of the AFM. They were also spread on flat quartz substrates. The cantilevers and substrates were then sintered during 3 h at 1150 °C, and they were subsequently cleaned in air plasma. The sintering leads to a firm attachment of the microparticles whereby they shrink to about 2.2 μm in radius.^44^

Forces between the microparticles were measured in a fluid cell with a closed-loop AFM (MFP-3D, Asylum Research) placed on an optical microscope (Olympus IX 73). The cantilever and the substrate are mounted in the fluid cell and the nanoparticle suspension is injected. A pair of microparticles is centered with the optical microscope and approach and retraction cycles are measured at a velocity of 500 nm s^−1^. The cantilever response is obtained by subtracting the baseline and the constant compliance region. The forces are calculated from the deflection through the spring constant of the cantilever. Their typical values were in the range of 0.2–0.5 N m^−1^ and they were determined from the thermal frequency response and the lateral dimensions of the cantilever as described by Sader *et al.*^45^ To determine the constant compliance region correctly, one must apply loads corresponding to a normalized force *F*/*R*_eff_ of at least 10 mN m^−1^. About 100 approach and retraction cycles were averaged to obtain the force profiles, which were subsequently block averaged. The resulting force resolution was about 2 pN and the distance resolution about 0.5 nm. Further details on similar force measurements can be found elsewhere.^9^

## Results and discussion

This study presents measurements of the thickness of the particle-free layer in aqueous suspensions of negatively charged nanoparticles near like-charged silica interfaces. These measurements are carried out with three independent experimental techniques, namely for the isolated interface with optical reflectivity and QCM, and for two interacting interfaces with direct force measurements based on the AFM. We will first discuss how these measurements are carried out with each of these techniques. Subsequently, the thickness measurements will be compared to the theoretical estimates presented above and to measurements available in literature. One type of silica nanoparticles and four different latex nanoparticles are used in this study, see Table 1. All these nanoparticles are negatively charged as verified by electrophoresis, see Table 2.

### Optical reflectivity

**Fig. 2:**
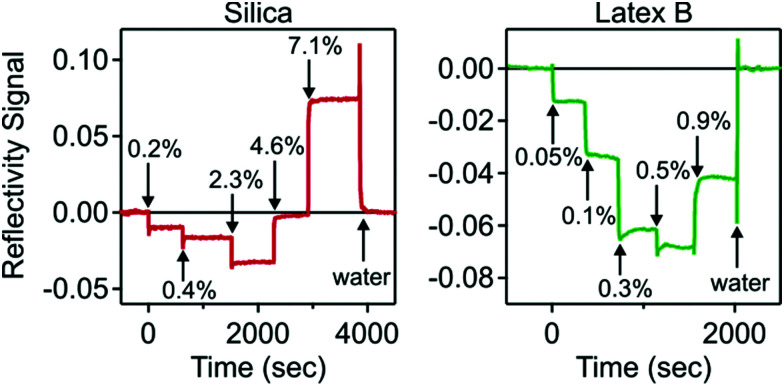
Experimental optical reflectivity traces of suspensions of charged nanoparticles near a like-charged silica interface. The arrows indicate different particle volume fractions injected. Silica nanoparticles are shown on the left, and latex nanoparticles on the right.

Different nanoparticle suspensions of increasing concentration were injected into the cell, and the reflectivity signal was recorded. Typical results for the silica and latex nanoparticles are shown in Fig. 2. One observes that for low concentrations the signal is always negative. At higher concentrations, the signal increases again, and for the silica particles it becomes positive again. Such a sign reversal is characteristic for optical matching, since in the reflectivity experiment the layer becomes invisible at a volume fraction around 5%. When the cell is flushed with pure water at the end of the experiment, the signal returns to zero, indicating that the process is entirely reversible. This type of response is rather unusual, since during particle deposition the signal is normally positive, and does not return to zero upon flushing.^28,29^

**Fig. 3:**
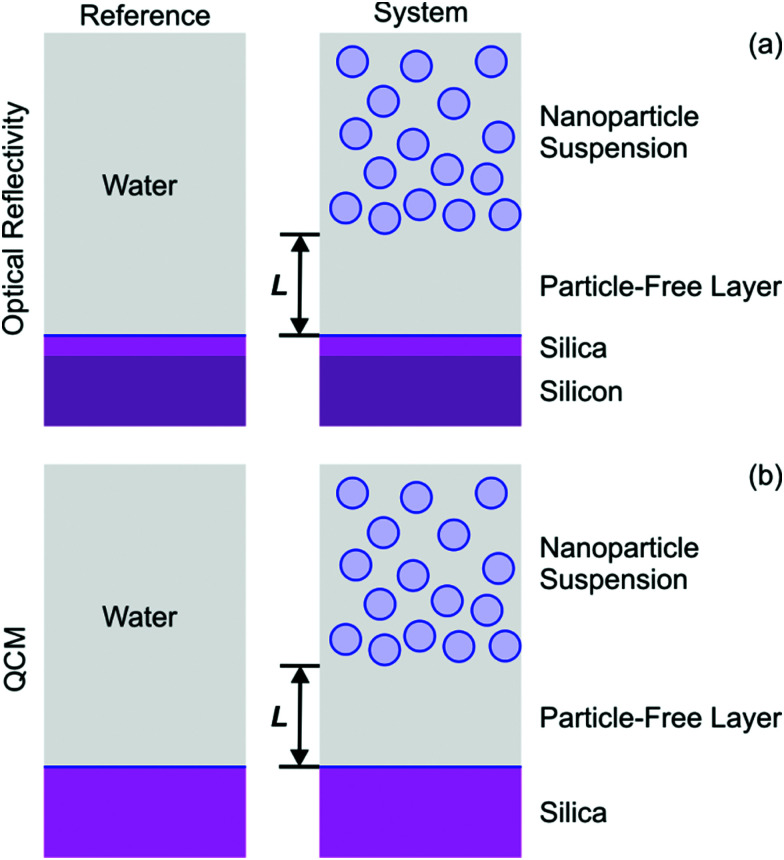
Schematic representation of the slab-models used to model the response of the isolated interface for (a) optical reflectivity and (b) QCM. The bulk suspension is assumed to be separated by a particle-free water layer of thickness *L*. The substrate is modeled as a silicon block with a layer of silica on its top in the case of optical reflectivity, while for QCM as a simple silica block. The model system used to interpret the measurements are shown on the right, and the reference with water in the bulk is shown on the left.

The observed signal can be explained by assuming the formation of a particle-free layer next to the interface. To interpret the data quantitatively we use an optical multi-layer model, see Fig. 3a. We assume that the substrate is separated from the particle suspension, which is the bulk liquid, by a particle-free water layer of thickness *L*. The substrate is modeled with a silicon block that is coated with a thin silica layer. For the silicon block and the silica layer the refractive indices are 4.132 + 0.033*i* and 1.461, respectively, as appropriate for the wavelength of 532 nm.^39,40^ Prior to the experiments, the thickness of the silica layer was determined for each substrate by ellipsometry in air, and typical values are 10–30 nm (see Experimental section). For the particle-free layer, we assume that the refractive index of pure water 1.335, while the refractive index of the silica suspension is obtained from the refractive index increment (RII) given Table 1. These calculations are implemented with the Abeles matrix method.^46^ The reflectivity of the 2-layer model is calculated with respect to the 1-layer reference model. The latter assumes that the silicon block together with the silica layer is in contact with bulk water, see Fig. 3a.

**Fig. 4:**
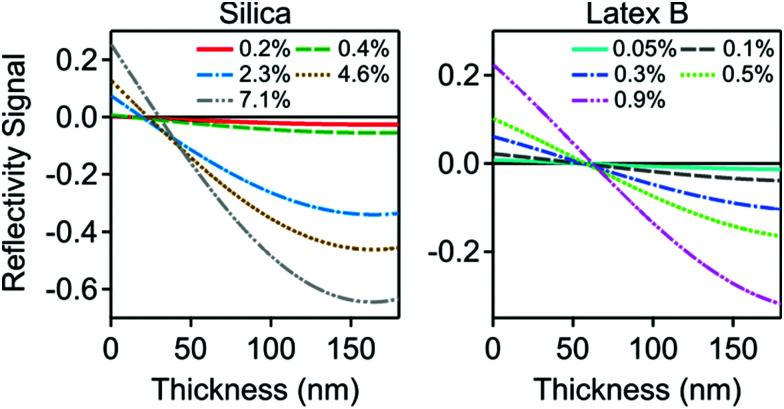
Calculated reflectivity signal *versus* the thickness of the particle free layer for the systems shown in Fig. 2. Silica nanoparticles are shown on the left, and latex nanoparticles on the right.

The only unknown parameter in the model is the thickness *L* of the particle-free layer. The reflectivity signal was calculated as a function of this thickness, and the results are shown in Fig. 4. One observes that a particle-free layer with a finite thickness is able to explain the negative reflectivity signal. The subsequent sign reversal is due to the fact that the thickness decreases with increasing particle concentration, as suggested by eqn (14). By inverting the calculated reflectivity signal *versus* the thickness shown in Fig. 4, one can extract the thickness from the experimental reflectivity signal. As the thickness of the particle-free layer increases, the signal goes though a minimum, and the inversion is no longer unique. This problem was particularly important for latex D. These measurements suggested that the particle-fee layer exceeds 400 nm, which becomes comparable to the wavelength of the light, and thus making the data analysis with a slab model questionable. For this reason, we do not report any optical reflectivity measurements for latex D. Another limitation of the reflectivity experiments is that too concentrated suspensions induce substantial light scattering from the suspension in the measurement cell, which makes the reflectivity signal weak and unreliable. The limiting volume fractions typically are 8% for the silica and 1% for the latex.

### Quartz crystal microbalance

**Fig. 5:**
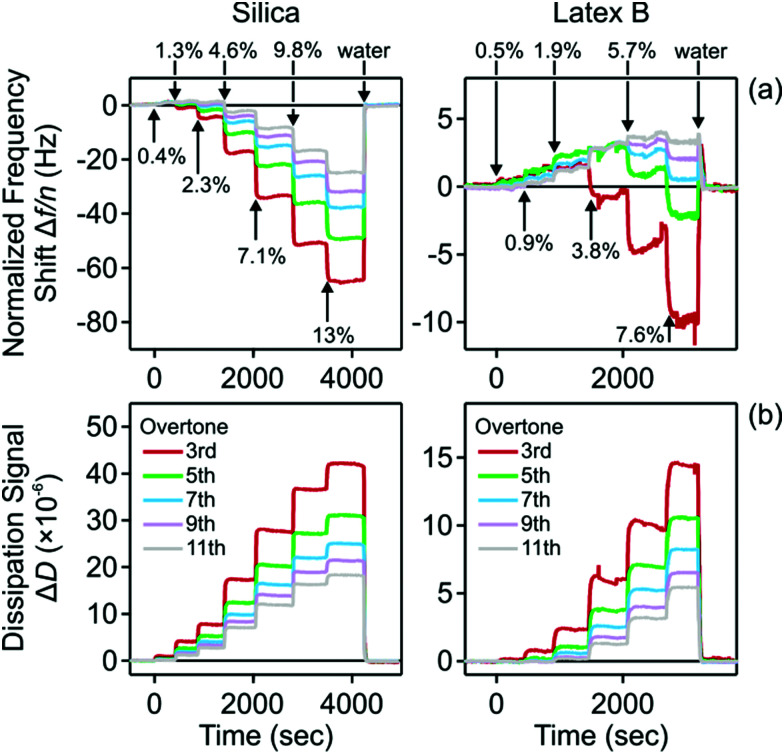
Experimental QCM traces of suspensions of charged nanoparticles near a like-charged silica interface. The different traces correspond to different overtones and the arrows indicate different particle volume fractions injected. (a) Normalized frequency shift and (b) dissipation signal. Silica nanoparticles are shown on the left, and latex nanoparticles on the right.

Similar experiments were carried out with the QCM. The cell was first flushed with water, and nanoparticle suspensions of increasing concentration were injected into the cell. Thereby, the normalized frequency shifts Δ*f*/*n* and dissipation signals Δ*D* were recorded for different overtones with time. The results for the silica and latex B are shown in Fig. 5. For the silica particles, the frequency shift decreases with increasing volume fraction, while the dissipation signals increase. For the latex nanoparticles, both parameters increase at low volume fractions. The frequency shift then goes though a maximum, and then decreases again. A similar behavior is observed for the silica particles, but the region where the frequency shift is positive is characterized by small values and low volume fractions, and is thus hardly visible on the scale of Fig. 5 (left). Such a sign reversal of the frequency shift may be interpreted as acoustic matching, whereby layer becomes invisible in the frequency response. However, the volume fraction, at which such matching occurs, is different for each overtone, and the dissipation signal keeps increasing. Therefore, the layer is never really invisible in the QCM experiment. For the larger particles, the dissipation signal also goes through zero and becomes negative, but this effect is only weak and hardly measurable. When the cell is being flushed with pure water, the signal returns to zero. This reversible behavior was observed in the reflectivity experiments too.

Experimental data are interpreted be means of an acoustic one-layer model.^42,47^ This model considers the particle-free layer as a Newtonian liquid. This layer is sandwiched between the bulk suspension and the silica coating of the quartz oscillator, see Fig. 3b. The quartz crystal is characterized by its density of 2.65 g cm^−3^ and the fundamental resonance frequency of *f*_0_ = 4.95 MHz. The particle-free layer is modeled as a layer with thickness *L* containing pure water with a density of 1.00 g cm^−3^ and a viscosity of *η*_0_ = 0.89 mPa s. The nanoparticle suspension is also modeled as a viscous fluid, which is also characterized by its density *ρ* and viscosity *η*. The density of the nanoparticle suspension is obtained from the density of the particles by means of the ideal mixing model. No-slip boundary conditions are used. The normalized frequency shift Δ*f*/*n* and dissipation signal Δ*D* are calculated by taking the real and imaginary part of the response function as described by Voinova *et al.*^47^ Thereby, one considers the differences between the response of the one-layer model and of the crystal in water, which is used as the reference, see Fig. 3b. Note that the expressions given by Voinova *et al.*^47^ use vacuum as the reference.

**Fig. 6:**
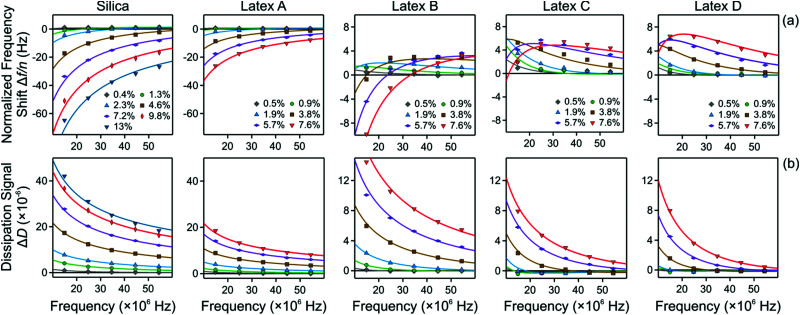
QCM response *versus* the oscillation frequency. The columns show different particle systems and the different curves various volume fractions as indicated. The points reflect the experimental values of the different overtones, and the solid lines best fits of the data with the slab model described in the text. (a) Normalized frequency shift and (b) dissipation signal.

The frequency dependence of the normalized frequency shifts Δ*f*/*n* and dissipation signals Δ*D* is now fitted simultaneously for all overtones with this model, which contains two adjustable parameters, namely the viscosity *η* of the nanoparticle suspension and the thickness *L* of the particle-free layer. The best fits of this model are presented as a function of the oscillation frequency *f* in Fig. 6. Note that this frequency is related to the overtone number *n* as *f* = *nf*_0_, where *f*_0_ is the fundamental resonance frequency. One observes that the fits are very good for all systems investigated, and they properly capture the unusual positive frequency shift and its maximum.

**Fig. 7:**
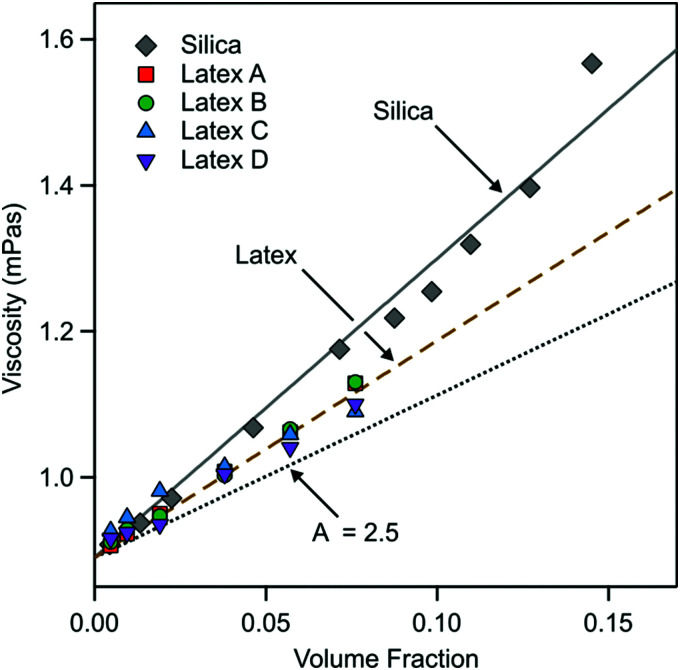
Viscosity of the nanoparticle suspension extracted from QCM experiments *versus* the particle volume fraction. The solid and dashed line is the best linear fits for silica and latex, respectively. The dotted line indicates the behavior for hard-spheres.

From these fits the viscosity of the nanoparticle suspension can be extracted. The results are shown in Fig. 7. The viscosity follows reasonably well the expected behavior^48–50^

17 *η* = *η*_0_(1 + *Aϕ*)

where *A* is a constant. For a suspension of hard spheres one has *A* = 2.5, while a larger value is expected for a suspension with charged particles due to the electroviscous effect. From the best fit of the experimental data shown in Fig. 7 we find *A* = 4.6 ± 0.1 for silica and *A* = 3.4 ± 0.1 for latex. These values agree rather well with previous measurements reported in the literature. For silica suspended in 3 mM NaCl and at pH 8.0 the value *A* = 4.2 was found,^48^ while the value of *A* = 4.4 was observed in a salt-free silica suspension.^49^ For salt-free sulfate latex suspensions the values of *A* = 3.8 were reported^21,50^ while the value of *A* = 4.7 for another latex.^50^

The quality of the fit of the QCM frequency response can be improved by adjusting the viscosity of the particle-free layer and by allowing for an elastic component of the particle suspension. Such generalized model was used to interpret the QCM response of a suspension of negatively charged latex particles near a silica interface by Helsing *et al.*^21^ We have found, however, that introducing these additional fitting parameters does not lead to any significant changes in the resulting layer thickness, but the fitting procedure becomes less stable. For this reason, we prefer to use the simpler model presented above.

**Fig. 8:**
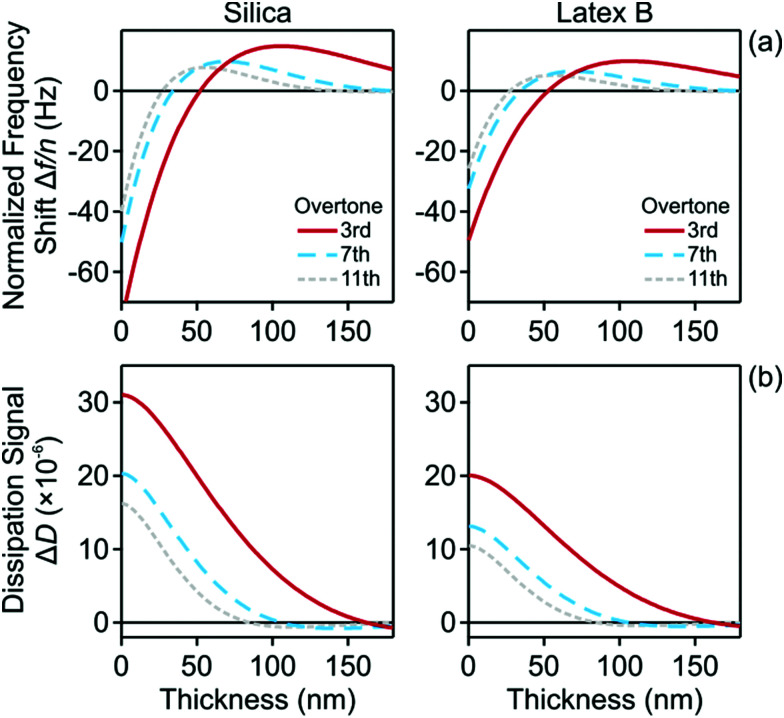
Calculated QCM signal *versus* layer thickness for silica nanoparticles at a volume fraction of 7.1% (left), and for latex B particles at a volume fraction of 7.6% (right). The different curves reflect different overtones as indicated. (a) Normalized frequency shift and (b) dissipation signal.

The QCM response can be better understood by considering the variation of the frequency shifts and of the dissipation signals with the thickness of the particle-free layer for different overtones. Such plots are shown in Fig. 8. One observes that the frequency shift increases at first, but then passes through zero (acoustic matching). For larger thickness, it goes through a maximum and then decreases again. The dissipation signals decreases with the thickness, becomes negative, and goes through a broad minimum at a larger layer thickness. This behavior is more pronounced for the lower overtones, and for those, the maximum in the frequency shift occurs for a larger layer thickness.

### Direct force measurements

**Fig. 9:**
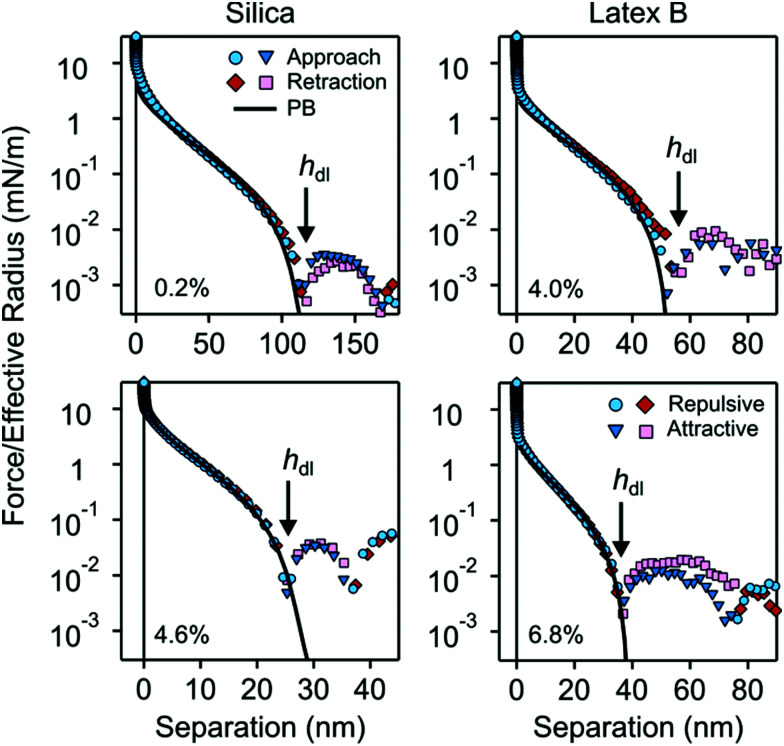
Semi-logarithmic representation of the normalized force profiles upon approach and retraction *versus* the surface separation between silica microparticles in nanoparticle suspensions of silica (left) and latex B (right). Attractive (positive) and repulsive (negative) forces are indicated separately. The respective volume fractions are indicated in each subfigure. The arrow indicates the thickness of the particle free gap *h*_dl_, which is identified with the first zero in the force curve. The thickness of the particle-free layer *L* is obtained from the relation *h*_dl_ = 2*L*.

Force profiles in nanoparticle suspension between pairs of larger silica microparticles were measured with the AFM. Typical magnitudes of the normalized force *F*/*R*_eff_*versus* surface separation *h* are shown in semi-logarithmic representation in Fig. 9. Due to limited force resolution, such measurements were only possible in suspensions of silica particles and for latex A and B.

At larger distances, one observes an oscillatory force profile, which is more pronounced for higher particle concentrations. This oscillatory profile reflects the layering of the nanoparticles between the microparticles, and originates from a liquid-like structuring in the bulk suspension. We will not discuss the oscillatory part of the force curve here, as it was analyzed by different authors in detail earlier.^2,4,7–10^

At smaller distances, the force profiles feature a strong repulsion. At very small distances, the force profiles decay slowly, and as one approaches the first zero in the profile, the decay becomes more rapid. This part of the force profile reflects the particle-free gap. Note the close similarly with theoretical pressure profile shown in Fig. 1d.

The small-distance part of the force profile was analyzed in two different ways. The thickness of the particle free layer was determined from the position of the first zero in the force curve *h*_dl_. This zero manifests itself as a minimum in the semi-logarithmic representation of the force as indicated with arrows in Fig. 9. The thickness *L* of the particle-free layer of the isolated interface is then determined with eqn (13).

The second way to analyze the force profile is by fitting with the PB model. The particle suspension is modeled as an asymmetric electrolyte, whereby the nanoparticles represent the multivalent coions. The parameters entering this description are the surface charge density of the microparticles, the corresponding regulation parameter, particle concentration, and the effective charge of the nanoparticles. The particle concentration is not being fitted, since its value is known from the suspension preparation. The remaining three parameters were obtained by least-squares fit of the force profiles to distances not exceeding their first zero. The agreement between the experimental force profiles and the ones calculated by means of PB theory is excellent, which provides further support for the validity of the PB theory in this region.

The regulation parameter obtained from these fits was found to fluctuate without any clear trends around the value of 0.68 ± 0.15 for the different particle suspensions investigated, as reported in an earlier study.^9^ For this reason, we have fixed the regulation parameter to the mean value and refitted all force curves. The resulting surface charge density of the microparticles was −11 ± 3 mC m^−2^ in the silica suspension and −5 ± 1 mC m^−2^ in both latex suspensions investigated. The latter values compare favorably with previous measurements in latex A suspensions,^9^ where a surface charge density of −6 mC m^−2^ was found. One should note that that study reported a larger surface charge density in a silica suspension, but such a discrepancy is expected due to different pH values of the respective suspensions.

The resulting magnitudes of the effective nanoparticle charge from the present force measurements were converted into a surface charge density, and the resulting values given in Table 2. These values compare reasonably well to the corresponding charge densities obtained by electrophoresis, which supports the consistency of the present description. Note that the value of −20 mC m^−2^ used in the PB calculations shown in Fig. 1 is the approximate average value of the charge densities given in Table 2.

### Thickness of the particle-free layer

**Fig. 10:**
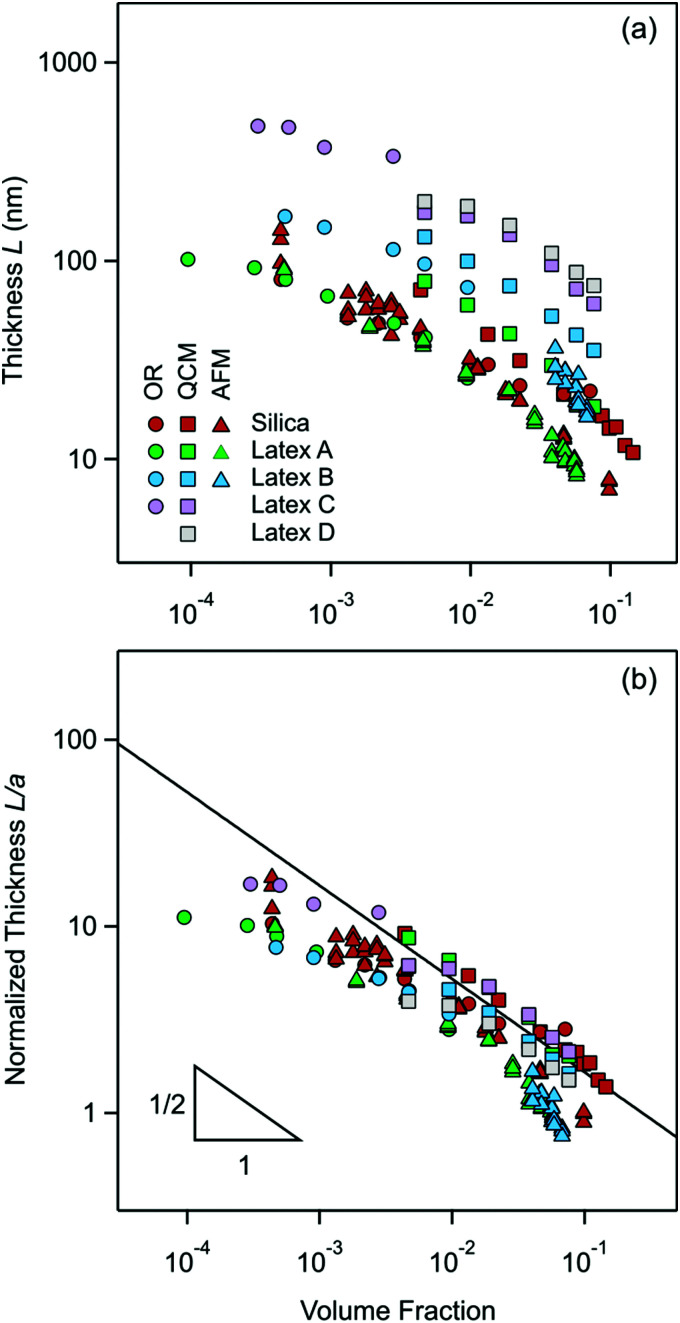
Present measurements of the particle-free layer thickness *versus* the volume fraction of nanoparticles as given in Table 1 with optical reflectivity (OR, circles), QCM (squares), and direct force measurements with the AFM (triangles). (a) Absolute values of the thickness and (b) normalized thickness with the particle radius. The solid line in (b) is the prediction of eqn (19).

The present measurements of the thickness *L* of the particle-free layer near an isolated interface are plotted *versus* the particle volume fraction in Fig. 10a. One observes that the layer thickness decreases with increasing particle concentration.

These thickness measurements were possible for the smaller particles with three independent techniques, namely reflectivity, QCM, and direct force measurements with the AFM. Therefore, it is instructive to compare the respective values. One observes that within the experimental scatter the values measured by reflectivity and AFM agree rather well, but the QCM measurements yield values about 30% larger.

One possibility for this discrepancy could be due to the assumed box-profile. In reality, the concentration profile of the particles beyond the particle-free layer decays to the bulk value in an oscillatory fashion. To investigate the influence of this effect, we have assumed the existence of an additional layer with a thickness of half the particle-free layer and a particle concentration that is 50% higher than in the bulk. When one introduces this modification into the analysis of the reflectivity and QCM data, one finds a larger layer thickness. This difference is typically 15% for the reflectivity, and about 25% for the QCM.

While this effect is not entirely negligible, the influence seems relatively low, and cannot explain the difference between the reflectivity and QCM measurements. The other possibility might be the effect of the substrate. While all substrates used are made of silica, their surface characteristics and roughness might be different. In particular, the substrate for reflectivity was a thermally grown silica layer, for QCM the silica layer was obtained by physical vapor deposition and in the force measurements sintered silica microparticles were used. Still another possibility might be the influence of the polydispersity of the nanoparticles used. At this point, it is difficult to pinpoint the origin of these disagreements in the layer thickness measurements. In spite of these minor discrepancies, however, the results obtained with the three different techniques are quite consistent, and they provide good estimates of the thickness of the particle-free layer.

The analytical pressure asymptote for the layer thickness given in eqn (14) can be reformulated in terms of the volume fraction. Moreover, all the particles used are highly charged, and for this reason their charge can be estimated under salt-free conditions with the corresponding saturation charge. The magnitude of this charge can be obtained from PB theory^51–53^18
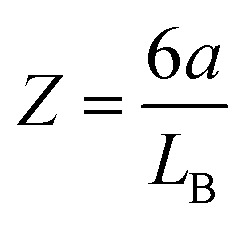
where *a* is the radius of the nanoparticles, and *L*_B_ is the Bjerrum length given by eqn (11). The saturation charge actually depends weakly on the volume fraction, but the value quoted in eqn (18) represent a good approximation in the relevant volume fraction range.^54,55^ This relation applies under salt-free conditions only, and larger values are expected in the presence of salt. Combining eqn (14) with eqn (15) and (18) the layer thickness can be expressed as19
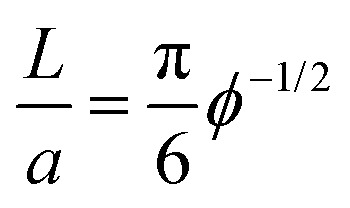
This pressure asymptote suggests that when the layer thickness normalized with the particle radius *L*/*a* is plotted *versus* the volume fraction *ϕ*, this plot should be universal and scale with  *ϕ*^−1/2^.

The present experimental measurements are represented in this fashion in Fig. 10b. In this representation, the data collapse relatively well indeed. Moreover, the data follow in a reasonable fashion the inverse square root concentration dependence predicted by eqn (19). The measurements for the silica particles with reflectivity and AFM are very close to this prediction, and the QCM data lie above as already remarked above. This agreement is also in line with earlier results obtained with similar silica particles, whereby the valence extracted from the force profile did agree rather well with the prediction of eqn (18).^9^ The layer thickness for the larger latex nanoparticles (latex C and latex D) also agree very well with eqn (19). On the other hand, the data for smallest latex A lie about factor of 2 below the prediction, while the data for latex B are in between. These discrepancies may have different explanations. The simplest explanation could be that the effective valence *Z* is larger than the value predicted by eqn (18). This deviation could be caused by the fact this equation is only valid in salt-free conditions, while traces of monovalent salt could be present. Such traces of salt would lead to larger values of *Z*.^52^ This aspect was already discussed for latex A in a previous publication in detail.^9^ The other possibility is that eqn (19) assumes that the charge density of the quasi-planar substrate is very high. When this assumption is no longer valid, meaning that the Gouy–Chapman length becomes comparable to the thickness of the particle free layer, a downturn in the layer thickness at higher volume fractions is observed. Such a downturn can be seen in the theoretical calculation shown in Fig. 1e, and also in the data for the smallest latex A. Finally, polydispersity may equally affect the resulting effective charge.^56^

Let us now compare these results with measurements that were published in the literature earlier. One should note that relatively few measurements of the particle-free layer thickness in nanoparticle suspensions were published. To our best knowledge, the first measurement was carried out by X-ray reflectivity in a suspension of silica nanoparticles near an isolated interface by Nygard *et al.*^11^ However, only one particle concentration was used. These measurements were recently completed for latex nanoparticles by QCM by Helsing *et al.*^21^ and by neutron reflectivity for silica nanoparticles by Maroni *et al.*^12^ Further measurements exist with direct force measurements in the slit-geometry for silica and latex nanoparticles by Scarratt *et al.*^9^ and for silica nanoparticles by Ludwig *et al.*^10^ The reported thickness of the particle-free gap as measured in direct force measurements is divided by a factor of two, as discussed above. The latter studies also report a variation the layer thickness with the particle concentration.

**Fig. 11:**
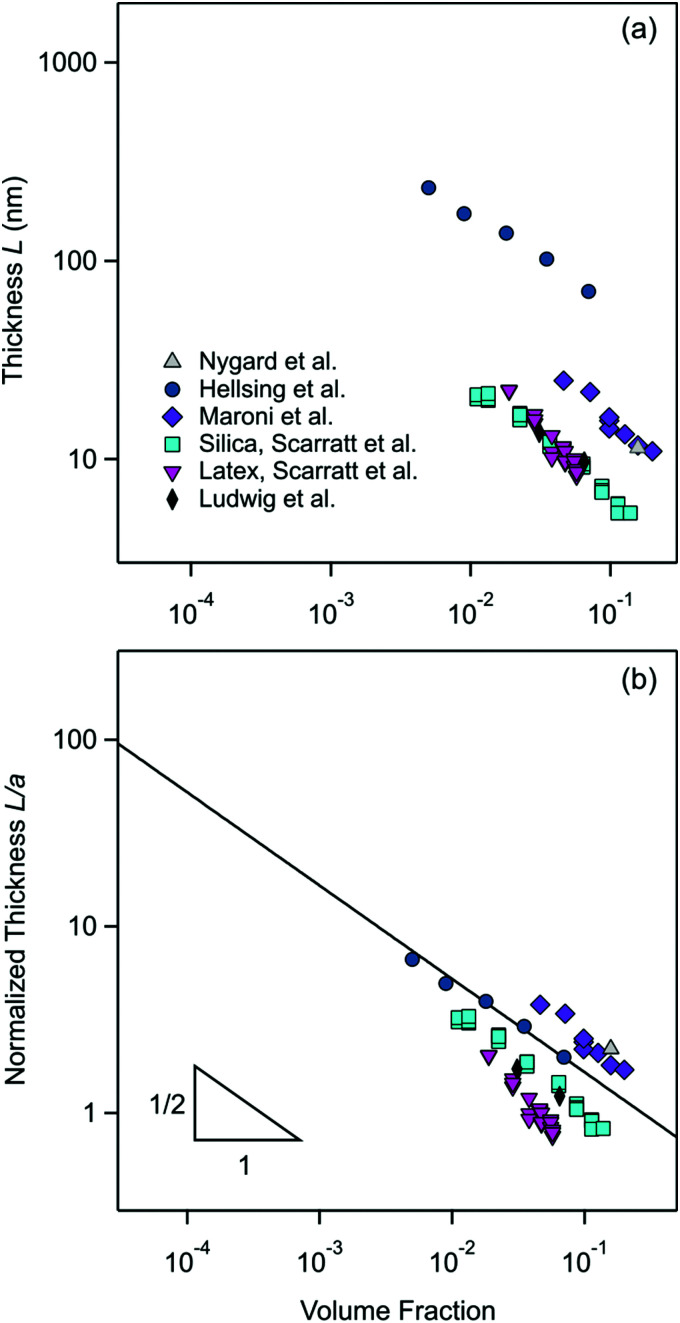
Earlier measurements of the particle-free layer thickness *versus* the volume fraction in salt free nanoparticle suspensions as reported in literature. (a) Absolute values of the thickness and (b) normalized thickness with the particle radius. The solid line in (b) is the prediction of eqn (19). More details concerning the system studied are given in Table 3.

The respective data are summarized in Fig. 11, whereby Table 3 provides additional details concerning the systems studied together with the respective references. While the available literature data are less complete than the ones presented here, they are also consistent with the pressure asymptote given in eqn (19). A notable discrepancy within the published data concerns the fact that the layer thickness obtained by neutron reflectivity by Maroni *et al.*^12^ is substantially larger than the one obtained by direct force measurements by Scarratt *et al.*^9^ The surprising aspect is that exactly the same particle suspension was used in these two studies. However, the silica substrates used in these studies were different. Maroni *et al.*^12^ used a naturally oxidized silicon block, while Scarratt *et al.*^9^ sintered colloidal microparticles. Interestingly, the results of neutron reflectivity agree well with X-ray reflectivity by Nygard *et al.*^11^ while the direct force measurements agree with the ones by Ludwig *et al.*^10^ At this point, the reason for this discrepancy is unclear to us.

**Table 3:** Measurements of particle-free layer thickness available in literature

Nanoparticles	Radius (nm)	Interface	Method	Ref.
Silica	5.2	Silicon wafer	X-Ray reflectivity	Nygard *et al.*^11^
Latex	35	Silica	QCM	Hellsing *et al.*^21^
Silica	6.5	Silicon block	Neutron reflectivity	Maroni *et al.*^12^
Silica	6.5	Silica microparticle	AFM colloidal probe	Scarratt *et al.*^9^
Latex	11	Silica microparticle	AFM colloidal probe	Scarratt *et al.*^9^
Silica	7.9	Silica micropaticle and silicon wafer	AFM colloidal probe	Ludwig *et al.*^10^

## Conclusion

The present study presents reliable thickness measurements of the particle-free layer in suspensions of charged nanoparticles next to a like-charged solid substrate. The reliability of the present measurements is asserted by the fact that three entirely independent experimental techniques provide very similar results for nanoparticles of different size and type. This thickness decreases with increasing particle concentration, and may vary between a few to several hundreds of nm. We further demonstrate that normalizing this thickness to the particle radius results in a universal inverse square root dependence on the volume fraction. This universal dependence can also be derived from PB theory.

The present study demonstrates that such measurements can be easily carried out with classical surface sensitive techniques, such as optical reflectivity and QCM. However, the data analysis must be adapted accordingly, as the experimental data feature unusual aspects, including optical and acoustic matching points. This aspect was already raised concerning QCM by Hellsing *et al.*,^21^ but not as yet for optical reflectivity. In our view, optical reflectivity turns out to the most suitable technique for such measurements available so far, and the data interpretation is much simpler than for the QCM. However, it has the disadvantage that it becomes unreliable for very thick layers, the least when the data are interpreted with the classical slab model, as we have done here.

The suitability of direct force measurements with the AFM to characterize this layer was already raised in a different context earlier by some of us.^19^ While direct force measurements provide additional information, they are surely more time-consuming to perform. Furthermore, we have managed to study only rather small nanoparticles with this technique. When comparing with the measurements with the surface sensitive techniques, one must realize that in the force profile one observes the particle-free gap between two surfaces in the slit geometry, while surface sensitive techniques probe the particle-free layer near an isolated interface, and therefore the former is larger by a factor of two. The presence of this factor of two can be also confirmed by comparing calculations with PB theory between the slit geometry and the isolated surface.

Obviously, the results obtained by these different techniques are not always identical, and sometimes they even differ by 30%. The most pronounced discrepancy is that QCM typically yields thicknesses of the particle-free layer that are larger than the ones obtained by the other techniques. Furthermore, the pressure asymptote that can be derived from PB theory is not always obeyed. While possible reasons why the experimental data disagree with the pressure asymptote can be put forward easily, we are currently unable to provide definitive explanations concerning the discrepancies between the results obtained by different experimental techniques. Their resolution remains an interesting task for future research.

## Conflicts of interest

There are no conflicts of interest to declare.
